# Detection of Hemorrhagic Transformation of Cerebral Infarcts with Ultra-low Field MRI

**DOI:** 10.1007/s00062-026-01666-0

**Published:** 2026-05-13

**Authors:** Dimah Hasan, Julian Sauer, Clara Heller, Annika Rieder, Konstantin Ueffing, Frederic de Beukelaer, Jörg Schulz, Johannes Schiefer, Manuel Dafotakis, Arno Reich, Martin Wiesmann, Florian Holtbernd, Charlotte Weyland

**Affiliations:** 1 Department of Neuroradiology, Universitätsklinikum Aachen, Aachen, Germany; 2 Department of Neurology, Universitätsklinikum Aachen, Aachen, Germany

**Keywords:** ULF-pMRI, Hemorrhagic transformation, Ischemic stroke, Diagnostic accuracy, Sensitivity

## Abstract

**Purpose:**

Hemorrhagic transformation (HT) within ischemic infarcts is a critical post-stroke complication that significantly influences secondary prevention strategies, particularly the timing and safety of anticoagulation. Ultra-low field (ULF) MRI (0.064 T, Swoop, Hyperfine) offers bedside neuroimaging, but its ability to detect hemorrhagic transformation compared to standard MRI (1.5 T or 3 T) and CT remains uncertain. This study aimed to evaluate the diagnostic accuracy of ULF-pMRI for detecting hemorrhagic transformation in ischemic stroke patients.

**Methods:**

We retrospectively analyzed 97 patients with ischemic infarcts who underwent both ULF-pMRI and standard neuroimaging (standard MRI or CT). The presence of hemorrhagic transformation was assessed by two independent readers (trained neuroradiologists) and by consensus reading in univariate analysis using the Heidelberg Bleeding Classification (HBC). Diagnostic accuracy metrics sensitivity, specificity, positive predictive value (PPV), and negative predictive value (NPV) were calculated with the standard method (standard MRI or CT within two days of ULF-pMRI) as the reference. Agreement was quantified using Cohen’s Kappa.

**Results:**

Of 97 stroke patients, *n* = 21 (21.6%) showed hemorrhagic transformation on standard imaging. Most findings corresponded to scattered small petechia and subarachnoid hemorrhage.

Reader 1 detected hemorrhagic transformation in ULF-pMRI with a sensitivity of 5/21 (23.8%), specificity 100%, positive predictive value (PPV) 100%, and negative predictive value (NPV) 82% and Reader 2 showed a sensitivity of 3/21 (14.3%), specificity 100%, PPV 100%, and NPV 81%, while consensus reading showed sensitivity of 3/21 (14.3%), specificity 100%, PPV 100%, NPV 81%. ULF-pMRI failed to detect petechial hemorrhages (0/13 HBC1a; 0/3 HBC1b), while detection improved for more extensive hemorrhagic transformations (HBC 1c and 3). After exclusion of petechial hemorrhages (HBC1a and HBC1b), ULF-pMRI detected 5 of 9 remaining hemorrhagic transformation subtypes (55.6%).

**Conclusion:**

Ultra-low-field MRI demonstrated low sensitivity for detecting hemorrhagic transformation in ischemic stroke, particularly for petechial hemorrhages (HBC class 1a and 1b). Detection was higher for non-petechial hemorrhagic transformations (HBC class 1c and 3), but overall performance remains insufficient for reliable therapy-guiding decisions.

## Introduction

Hemorrhagic transformation (HT) of acute ischemic stroke (AIS) is frequent and often asymptomatic but can lead to neurological deterioration and worse clinical outcomes [1, 2]. Reliable detection of HT is important for guiding the initiation of antithrombotic therapy, including antiplatelet therapy and oral anticoagulation. Although current randomized trials suggest that an early start of direct oral anticoagulants in patients with mild to moderate infarcts without significant HT is safe and does not increase symptomatic intracranial bleeding, the optimal timing of antithrombotic therapy in patients with large infarcts complicated by HT remains uncertain [3–5].

Non-contrast computed tomography (CT) remains the standard of care for the detection of intracerebral hemorrhage (ICH) due to its broad availability and high sensitivity for acute blood. However, multiple studies have reported comparable sensitivity of magnetic resonance imaging (MRI) for the detection of hemorrhage and have further demonstrated its superiority over CT in identifying early ischemic changes and differentiating subtypes of hemorrhage [6–9].

Ultra-low field (ULF) MRI (0.064 T, Swoop, Hyperfine) offers advantages for critically ill patients, as it enables bedside neuroimaging without the need for transporting unstable patients to a conventional standard MRI suite or CT [10]. Recent data suggest that low-field portable MRI can reliably detect intracerebral hemorrhage, supported by advances in image reconstruction and acquisition techniques. These findings highlight the potential of portable MRI for rapid, point-of-care neuroimaging [11, 12]. However, its ability to detect hemorrhagic transformation in ischemic stroke compared to standard MRI (1.5 T or 3 T) and CT remain uncertain.

This study aimed to evaluate the diagnostic accuracy of ULF-pMRI for detecting hemorrhagic transformation in ischemic stroke patients.

## Methods

### Study Design and Participants

This was a single-center retrospective analysis conducted at a tertiary university hospital *(University Hospital RWTH Aachen)*, based on ULF-pMRI imaging with clinical indication. We included all patients who underwent ULF-pMRI (0.064 T, Hyperfine MRI device) imaging for the evaluation of ischemic infarction between May 2025 and October 2025 in the neurological stroke unit or intensive care unit as part of routine clinical care. During their hospital stay, all patients underwent a standard imaging examination, with additional imaging such as conventional CT or MRI performed depending on their clinical condition and specific indications. Clinical data, including in-hospital antithrombotic therapy, were extracted from medical records. In-hospital antithrombotic therapy was defined as initiation of treatment prior to ULF-pMRI acquisition; accordingly, ULF-pMRI was performed after therapy initiation in most cases.

### Ethics Approval and Consent to Participate

This retrospective analysis was approved by the ethics committee, medical faculty, University Aachen, (local registration number: 25-306) and complies with the Declaration of Helsinki for Medical Research involving human subjects. Written informed consent was waived for this analysis due to the retrospective nature of the study design. The study was conducted according to the Strengthening the reporting of observational studies in epidemiology (STROBE) criteria [13].

### ULF-pMRI and Scan Protocol

The 0.064 T Swoop® Portable MR Imaging System (Hyperfine, Inc., Guilford, CT, USA; hardware version 1.7; software version 8.7 (commercial)) was used for all patients reported in this study. The protocol used for all patients was the standard scan protocol provided by Hyperfine: Axial FLAIR (inversion-recovery turbo spin echo, acquisition time = 7 min 50 s, resolution = 1.7 × 1.7 × 5 mm, TE/TR/TI = 170/3000/1290 ms, receiver bandwidth = 64 kHz, echo train length = 68, excitation/refocusing/inversion flip angles = 90°/180°/180°) and axial diffusion-weighted imaging and Apparent Diffusion Coefficient (ADC) Maps (turbo spin echo, acquisition time = 8 min 40 s, resolution = 2.4 × 2.4 × 6 mm, TE/TR = 62/850 ms, receiver bandwidth = 52 kHz, echo train length = 44, excitation/refocusing flip angles = 90°/180°). Axial T1-weighted imaging (turbo spin echo, acquisition time = 4 min, resolution = 1.6 × 1.6 × 5 mm, TE/TR/TI = 5.4/880/322 ms, receiver bandwidth = 64 kHz, echo train length = 32, excitation/refocusing flip angles = 90°/180°). Axial FAST T2-weighted imaging (turbo spin echo, acquisition time = 2 min 10 s, resolution = 1.6 × 1.6 × 5 mm, TE/TR = 195/2000 ms, receiver bandwidth = 64 kHz, echo train length = 80, excitation/refocusing flip angles = 90°/180°).

### ULF-pMRI Image Post-processing

Image reconstruction and post-processing were performed using the manufacturer’s proprietary pipeline. Reconstruction included noise suppression, T2 decay compensation, apodization filtering, and advanced gridding with phase-sensitive parallel imaging. Deep learning-based components were applied for reconstruction and denoising, including learned methods to compensate for undersampling and convolutional neural network-based noise reduction to improve signal- and contrast-to-noise ratio. Post-processing further included geometric distortion correction, intensity (bias field) correction, and additional denoising. These steps improve overall image quality but do not compensate for motion or external artifacts.

### Conventional Imaging

All conventional MRI examinations were performed on a 1.5 T system (MAGNETOM Aera, Siemens Healthineers, Erlangen, Germany) using a standardized clinical protocol. This protocol included axial T2*-weighted imaging (2D gradient echo sequence, slice thickness 3 mm), FLAIR imaging, and diffusion-weighted imaging with ADC maps. Additional sequences were performed depending on the clinical context. CT examinations were performed using either a 40-slice scanner (SOMATOM AS, Siemens Healthineers) or a photon-counting CT system (NAEOTOM Alpha, Siemens Healthineers), following standard clinical protocols.

Study endpoints were the sensitivity and specificity of ULF-pMRI in detecting hemorrhagic transformation in ischemic brain infarction.

### Radiological Assessment

For radiological assessment, two experienced neuroradiologists (Reader 1 with 13 years of experience and Reader 2 with 11 years of experience), blinded to each other’s evaluations, independently reviewed all ULF-pMRI images across all four sequences. Subsequently, they also assessed the reference standard examinations in a blinded manner with respect to the presence or absence of infarct demarcation and hemorrhagic transformation, according to the Heidelberg classification [14]. To establish a reference standard, a consensus reading was performed, ensuring uniform results for further analysis. Patients with CT performed with one day after DSA/endovascular procedures showing subarachnoid hyperdensities were excluded from the primary analysis due to the potential for contrast staining mimicking hemorrhage [15]. Cases with hemorrhage confirmed by standard MRI were retained.

All images were reviewed on a standard PACS workstation.

### Statistics

Data are shown as number of events and percentage (*n*, %) and median with interquartile range (IQR) after testing for normal distribution with the Kolmogorov Smirnov test or Shapiro-Wilk Test. Diagnostic accuracy metrics: sensitivity, specificity, positive predictive value (PPV), and negative predictive value (NPV); were calculated with the standard method (standard MRI or CT) as the reference. Interrater agreement was assessed using Cohen’s Kappa. All tests were two-sided and a *p*-value of < 0.05 considered statistically significant. Statistical analyses were performed with SPSS Statistics (29.0; IBM, Armonk, NY). Analysis was based on one ULF-pMRI scan per patient.

## Results

We retrospectively reviewed 125 patients who underwent ULF-pMRI examination for ischemic stroke between May 2025 and October 2025. Of these, 24 patients were excluded due to incomplete ULF-pMRI examination or lack of comparable standard imaging. Among these excluded cases, six patients had CT examinations performed shortly after DSA/endovascular procedures showing positive findings (two with isolated subarachnoid hyperdensities and four with subarachnoid hyperdensities associated with additional parenchymal hyperdensities). Given the difficulty in distinguishing contrast staining from true hemorrhage in this setting, these cases were not included in the final analysis.

**Fig. 1:**
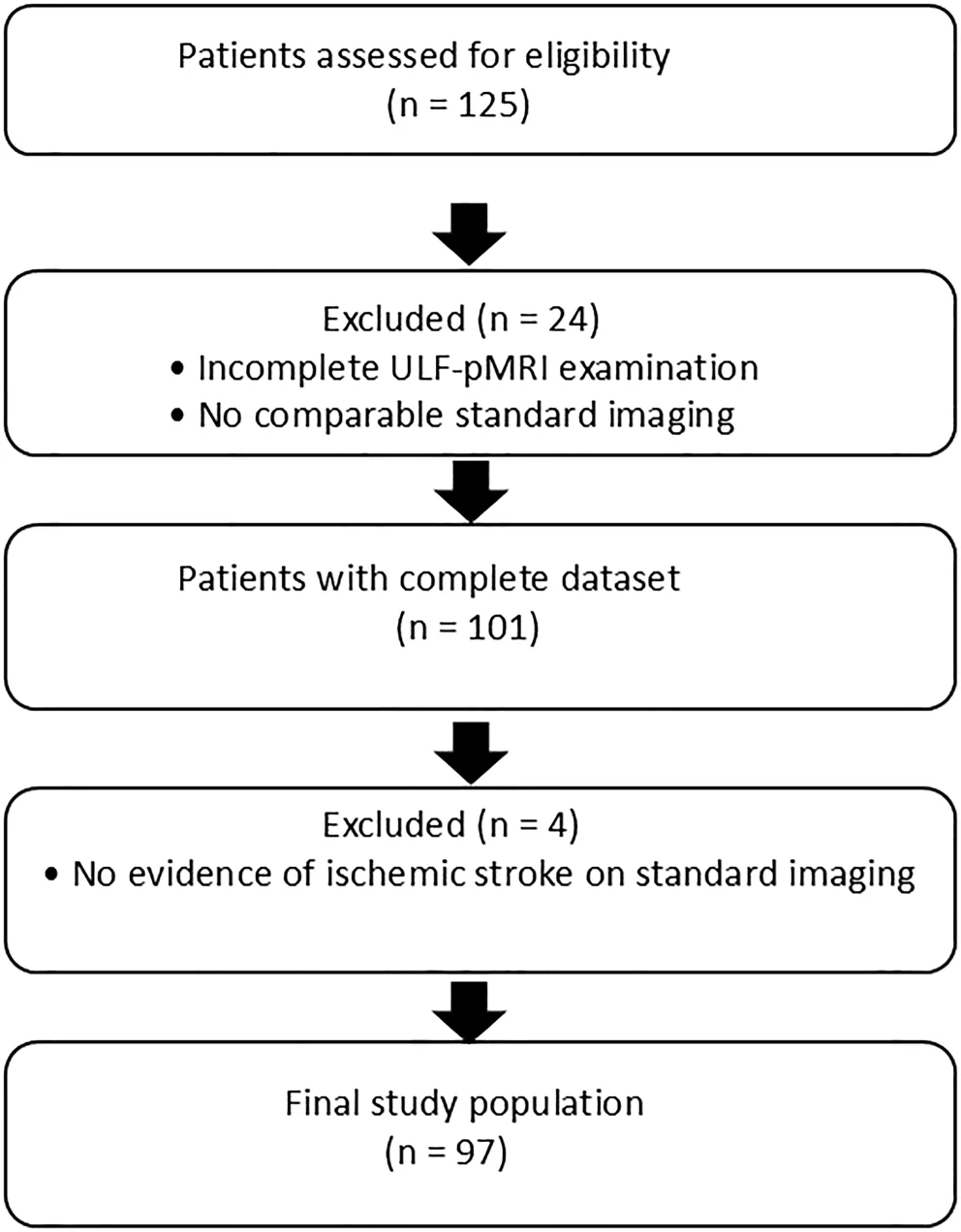
Flowchart of patient inclusion and exclusion

A total of 101 patients remained. Among these, four did not show evidence of ischemic stroke on standard imaging and were therefore excluded from further analysis. The final study population consisted of 97 patients. The patient selection process is summarized in Fig. 1.

The 97 patients had corresponding imaging performed during their hospital stay, with a median interval of 1 day. In most cases (*n* = 73, 75.3%) this was CT imaging, and in 24 cases (24.7%) standard MRI. Study population characteristics are summarized in Table 1.

**Table 1:** Baseline Characteristics and Treatment Details of the Study Population (*N* = 97)

Demographics and Clinical Characteristics	
Age, median (IQR)	75 (62–84)
NIHSS score, median (IQR)	5 (2–19.0)
*Acute and Antithrombotic Treatment*	*n (%)*
Mechanical recanalization	43 (44.3)
Intravenous thrombolysis	33 (34.0)
Antiplatelet therapy, prehospital	30 (30.9)
Oral anticoagulation, prehospital	18 (18.6)
Antiplatelet therapy, in-hospital	28 (28.9)
Anticoagulation, in-hospital	40 (41.2)

Of those cases, *n* = 21 (20.64%) showed hemorrhagic transformation, which was defined according to the Heidelberg bleeding classification (HBC) [14].

Of these cases, hemorrhagic transformation was detected in 16/21 cases (76.2%) on CT and in 5/21 cases (23.8%) on standard MRI. The distribution of hemorrhagic transformation subtypes according to the Heidelberg bleeding classification (HBC) was as follows: scattered small petechiae (HBC1a: *n* = 13, 61.9%), subarachnoid hemorrhage (HBC3c: *n* = 7, 33.3%), confluent petechiae in ischemic tissue (HBC1b: *n* = 3, 14.3%), larger hemorrhage without substantial mass effect (HBC1c: *n* = 1, 4.7%), and intraventricular hemorrhage (HBC3b: *n* = 1, 4.7%), with a total of 25 hemorrhagic transformation subtypes classified across 21 cases, as multiple subtypes could occur in a single patient. A detailed breakdown of hemorrhagic transformation subtypes according to the Heidelberg bleeding classification (HBC), including corresponding ULF-pMRI detection rates and sensitivities, is presented in Table 2.

**Table 2:** Distribution of hemorrhagic transformation subtypes according to the Heidelberg bleeding classification (HBC) and corresponding ULF-pMRI detection rates. Different bleeding subtypes in the same patient were observed in several cases; therefore, totals refer to subtype counts rather than individual patients. Sensitivity estimates for subtypes with very small sample sizes (e.g., *n* = 1) should be interpreted with caution.

HBC Subtypes	Total (*n*)	CT (*n*)	Standard MRI (*n*)	Detected by ULF-pMRI (*n*)	Sensitivity for ULF-pMRI
HBC1a	13	10	3	0	0/13 (0%)
HBC1b	3	2	1	0	0/3 (0%)
HBC1c	1	0	1	1	1/1 (100%)
HBC3b	1	1	0	1	1/1 (100%)
HBC3c	7	6	1	3	3/7 (42.9%)
Total	25	19	6	5	5/25 (20%)

When stratified by HBC subtype, ULF-pMRI showed no detection of petechial hemorrhages (HBC1a: 0/13; HBC1b: 0/3), while detection was higher for less frequent but more extensive hemorrhagic transformations, including HBC1c (1/1), HBC3b (1/1), and HBC3c (3/7). These estimates should be interpreted with caution due to the very small sample sizes.

After exclusion of petechial hemorrhages (HBC1a and HBC1b), ULF-pMRI detected 5 out of 9 remaining hemorrhagic transformation subtypes (55.6%). These findings occurred in 7 patients, of whom 3 were positive on ULF-pMRI.

With regard to clinical relevance, no cases of symptomatic hemorrhagic transformation with probable relatedness (HBC class 2) were observed in our cohort. Among asymptomatic hemorrhagic transformations, ULF-pMRI detected 5/12 (42%) cases classified as having possible relatedness (HBC classes 1b, 1c, and class 3 hemorrhages). In contrast, hemorrhagic transformations with unlikely relatedness (HBC class 1a) were not detected by ULF-pMRI (0/13).

In the subgroup of patients with CT as the reference standard, ULF-pMRI detected 2 out of 16 hemorrhagic transformations (12.5%).

Reader 1 detected hemorrhagic transformation on ULF-pMRI in 5 out of 21 cases, yielding a sensitivity of 5/21 (23.8%) and specificity of 100%, indicating that only a small proportion of hemorrhagic transformations identified on standard MRI/CT were detected using ULF-pMRI, with absence of false-positive findings. The positive predictive value was 100%, indicating that all HTs identified on ULF-pMRI were confirmed by standard imaging, whereas negative predictive value was 80% showing that most negative results were accurate. Table 3.

**Table 3:** Diagnostic performance of ULF-pMRI for the detection of hemorrhagic transformation compared with standard imaging

Reading	True Positive (TP)	False Negative (FN)	False Positive (FP)	True Negative (TN)
*Reader 1*	5	16	0	76
*Reader 2*	3	18	0	76
*Consensus*	3	18	0	76

Reader 2 detected hemorrhagic transformations on ULF-pMRI in 3 out of 21 cases, resulting in a low sensitivity of 3/21 (14.3%) and a specificity of 100% with a positive predictive value of 100% and a negative predictive value of 83% Table 3.

Consensus reading showed a similar low sensitivity of 3/21 (14.3%) and high specificity of 100%. The positive predictive value was 100% based on very few positive calls, and the negative predictive value was 81%. Table 3.

Agreement with the standard imaging was low but statistically significant; The McNemar test demonstrated a significant difference between ULF-pMRI and the standard method (*p* < 0.001), driven predominantly by false negative cases and reflecting systematic underdetection of hemorrhagic changes as assessed by ULF-pMRI.

Interrater agreement between the two readers was good for ULF-pMRI, with a Cohen’s kappa of κ = 0.740 (*p* < 0.001) and similarly high for standard imaging with a Cohen’s kappa of κ = 0.722 (*p* < 0.001).

In detail, the consensus evaluation found hemorrhagic transformation in the ULF-pMRI in three patients.

**Fig. 2:**
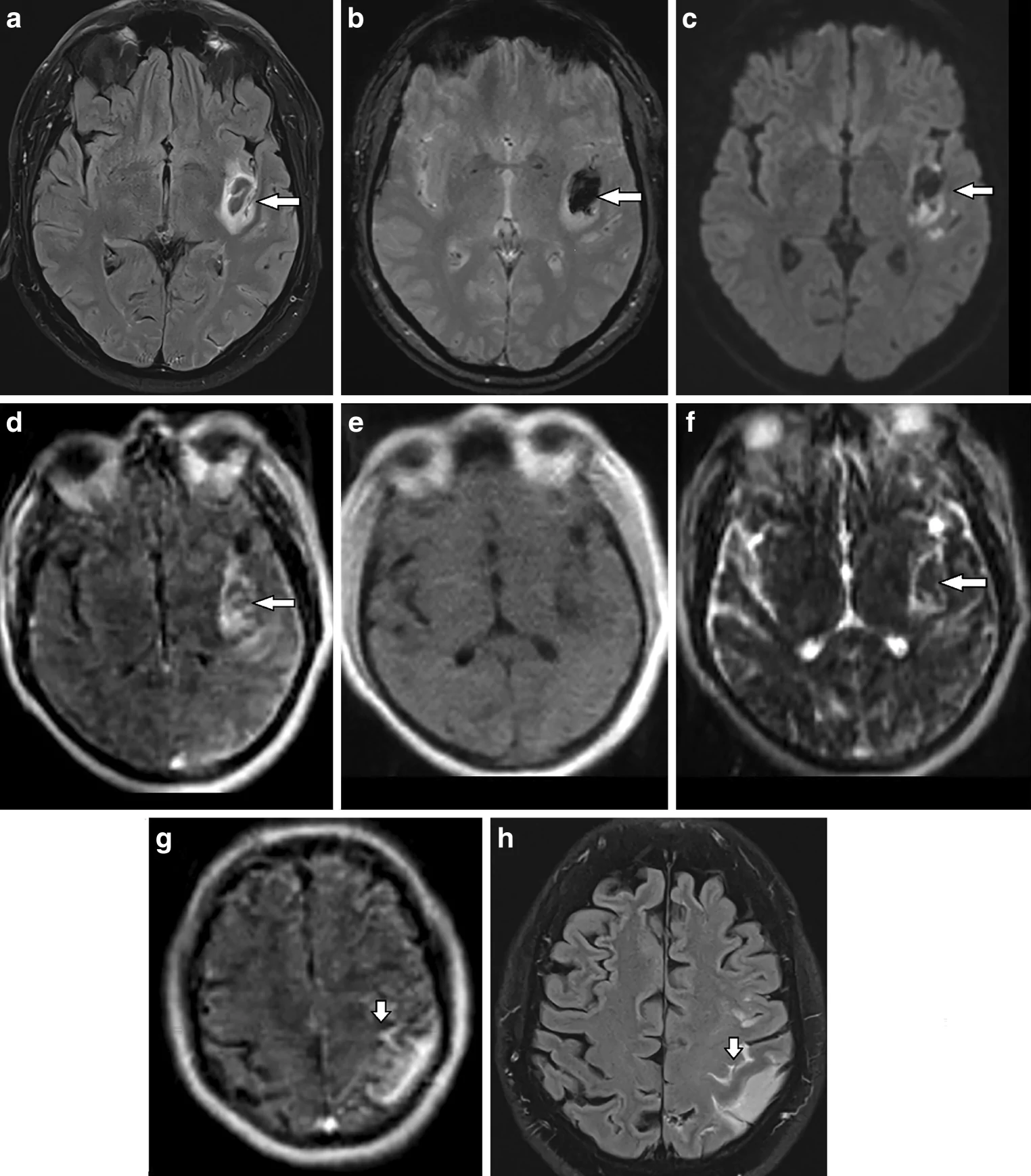
A 59-year-old patient with left in the middle cerebral artery territory ischemia due to M2 occlusion showing an early subacute infarction involving the left insula with hemorrhagic transformation and associated subarachnoid hemorrhage. ULF-pMRI demonstrated a large left insular parenchymal hemorrhage without significant mass effect (HBC 1c; arrows in **a**–**c**: Standard MRI, **d**–**f**: ULF-pMRI) and widespread subarachnoid hemorrhage (HBC3c) extending to the right central sulcus, visible as FLAIR hyperintensities (arrows in **g** and **h**). Standard MRI was acquired one day after ULF-pMRI

**Fig. 3:**
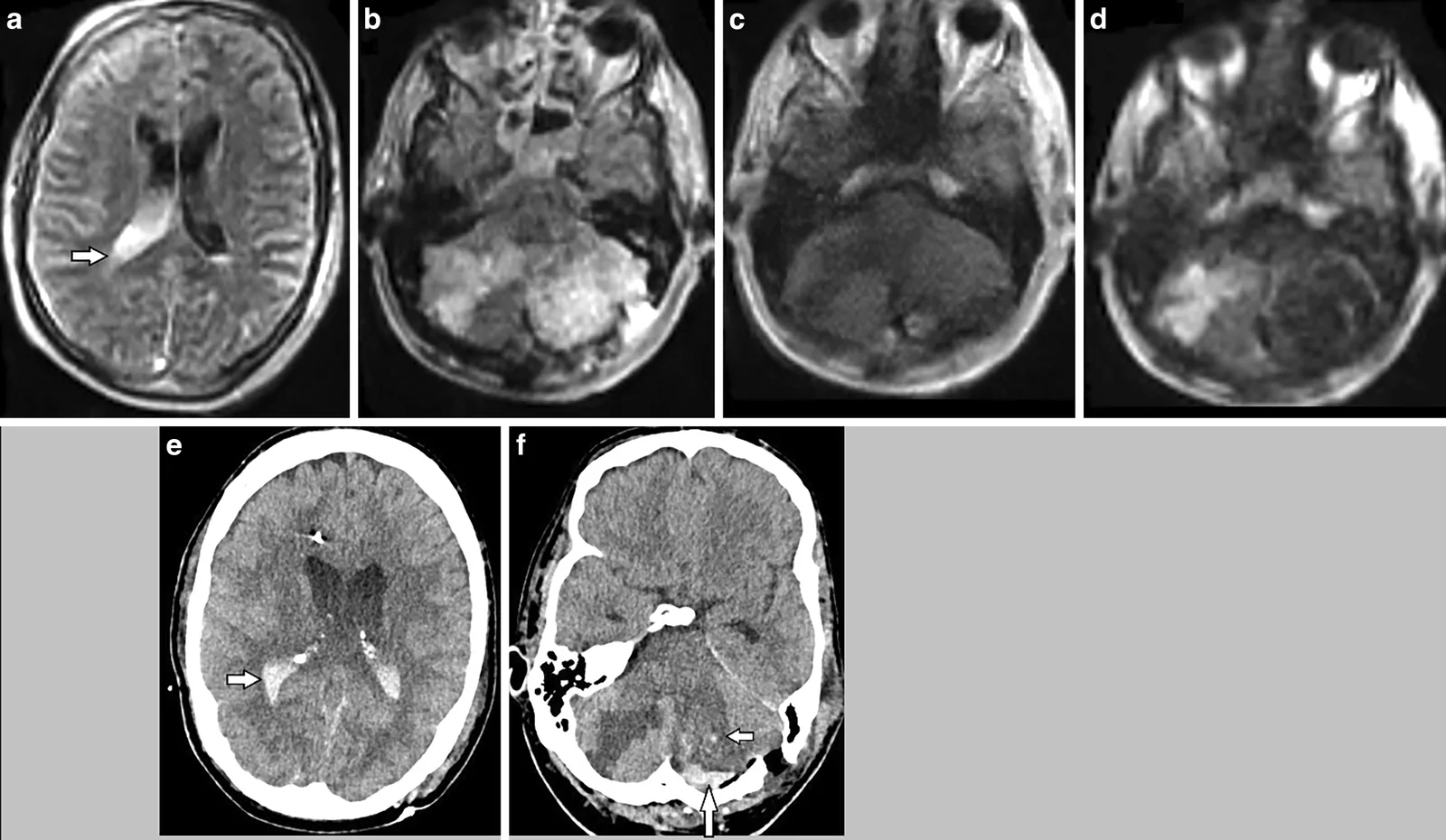
**a** 57-year-old patient with bilateral cerebellar infarctions with hemorrhagic transformation. ULF-pMRI showed massive intraventricular hemorrhage (HBC3b), predominantly in the right lateral ventricle (arrow in **a** FLAIR ULF-pMRI; **e** CT). Hemorrhagic components were clearly visible only on CT, which was obtained after surgical decompression for edema and impending herniation. Follow-up SWI MRI was performed one day later (infarct age: 4 days)

In the first patient, the ULF-pMRI revealed a large parenchymal hemorrhage without significant mass effect (HBC 1c) accompanied by subarachnoid hemorrhage (HBC3c). In this case the subarachnoidal hemorrhage was not limited to the area near the infarct but showed a more widespread distribution. Figure 2. In the second patient, ULF-pMRI revealed intraventricular hemorrhage (HBC3b) with accompanying subarachnoid hemorrhage (HBC3c). Figure 3. In the third patient we noticed diffuse subarachnoid hemorrhage in the basal cisterna and not just adjacent to the infarct.

**Fig. 4:**
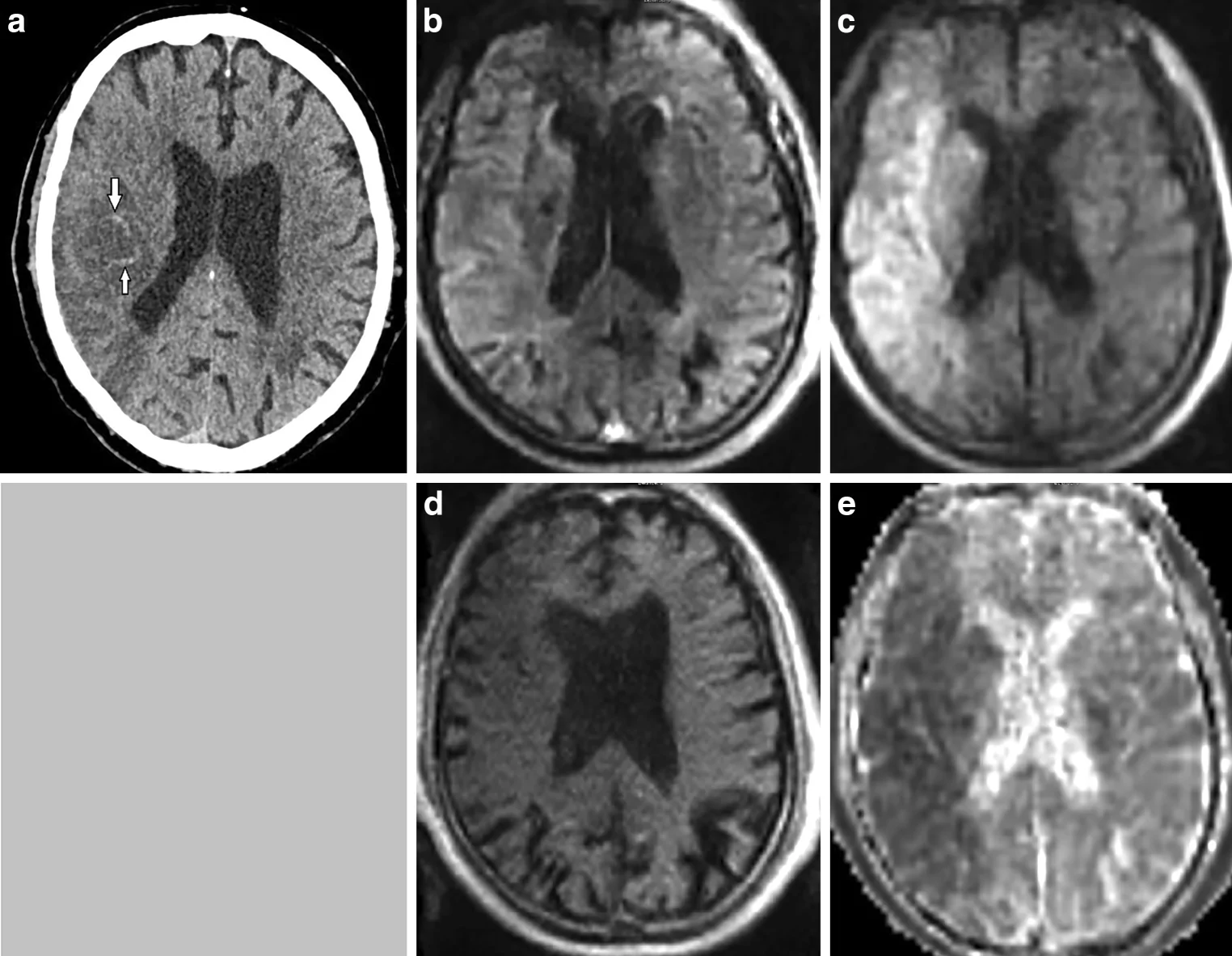
Hemorrhagic transformation (HBC1a) within the extensive subacute left-sided middle cerebral artery territory infarction was detected on CT (arrows in **a**) but was not visible on ULF-pMRI (**b**–**e**)

**Fig. 5:**
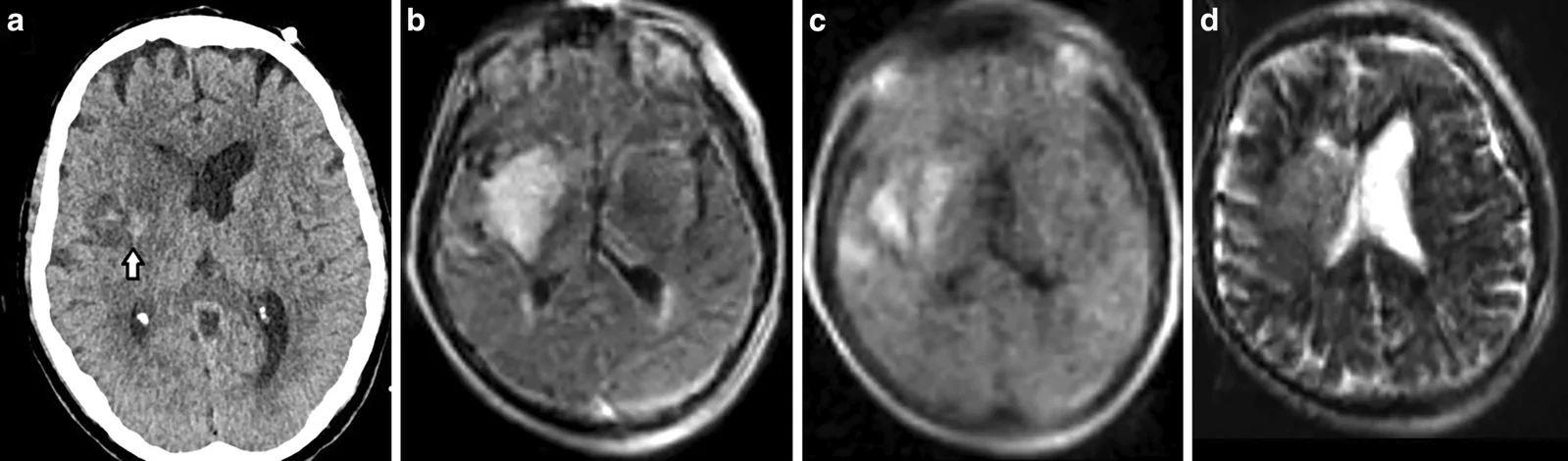
Hemorrhagic transformation (HBC 1b) was detected on CT (arrows in **a**) but was not visible on ULF-pMRI (**b**–**d**) in a right basal ganglia infarction following occlusion and subsequent recanalization of the right middle cerebral artery. CT and MRI were performed one day later in a 65-year-old patient

In contrast, subarachnoid hemorrhages that were limited to the area adjacent to the infarct were not detected in ULF-pMRI. Similarly, petechial hemorrhagic transformations (HBC 1a and HBC 1b) were not also visualized in ULF-pMRI. Figures 4 and 5.

## Discussion

In this study, ULF-pMRI demonstrated low sensitivity (14.3%) but high specificity for detecting hemorrhagic transformation (HT) in ischemic stroke. Importantly, this limited sensitivity was primarily driven by the inability of ULF-pMRI to detect petechial hemorrhages, as demonstrated by the absence of detection in HBC 1a (0/13) and HBC 1b (0/3) subtypes.

In our cohort, hemorrhagic transformation was detected by conventional imaging in 20.6% of ischemic stroke patients and served as the reference standard. This prevalence is consistent with previously published data reporting HT in approximately 25–30% of acute ischemic strokes [1, 2, 16, 17].

The limited sensitivity of ULF-pMRI for detecting HT can be explained by technical constraints inherent to the ultra-low field MRI system. MRI, especially susceptibility-based sequences such as GRE and SWI, is more sensitive than CT in detecting hemorrhagic transformations due to its ability to visualize paramagnetic blood products, enabling detection of small or early hemorrhages that may not be visible on CT [7, 18–20].

The ULF-pMRI lacks susceptibility-based imaging, making hemorrhage detection relies on conventional T1-weighted, T2-weighted, and FLAIR sequences. These sequences have limited sensitivity for detecting hemorrhagic transformation within infarcted tissue, particularly in the acute phase and in cases of petechial hemorrhage [21]. In line with this, our subgroup analysis showed that ULF-pMRI did not detect any petechial hemorrhages (HBC1a and HBC1b), while detection was better for more extensive hemorrhagic transformations and bleeding remote of infarcted brain tissue (HBC class 1c and 3). In total, 5 out of 9 hemorrhagic transformation subtypes (55.6%) were identified in HBC class 1c and 3, corresponding to 3 out of 7 affected patients.

Future improvements in ULF-pMRI may arise from advances in image reconstruction, particularly through deep learning-based approaches that enhance contrast-to-noise ratio (CNR) and reduce noise. However, conventional susceptibility-based MRI techniques are fundamentally limited at ultra-low field MRI due to reduced susceptibility effects. Alternative strategies, including optimized sequence design or inversion-recovery-based techniques targeting hemorrhagic components, may offer potential for improving hemorrhage detection and warrant further investigation.

Our findings differ from those of Mazurek et al., who reported higher detection rates of intracerebral hemorrhage using ULF-pMRI [11, 12]. This discrepancy is most likely explained by differences in study populations, as their cohort included patients with primary intracerebral hemorrhage or space-occupying hematomas, which are more readily detectable. In contrast, our study focused exclusively on hemorrhagic transformations within ischemic infarcts, a pathology that is inherently more challenging to detect without susceptibility-based imaging.

Another important factor to consider is the time interval between ULF-pMRI and the reference standard imaging (CT or standard MRI). Hemorrhagic transformation is a dynamic process that may evolve over time, and differences in time periods between imaging may therefore contribute to discrepancies in detection [22]. In our cohort, only 5 of the 21 patients with hemorrhagic transformation underwent reference imaging on the same day, whereas the remaining patients had follow-up imaging with a median delay of 1 day. In particular, hemorrhagic changes may develop or become more apparent on follow-up imaging, potentially leading to an underestimation of ULF-pMRI performance when compared to later reference imaging. This aspect should be taken into account when interpreting the observed concordance between modalities.

Beyond the specific setting of hemorrhagic transformation, these findings raise important questions regarding the role of ULF-pMRI in other clinical scenarios where hemorrhage detection is critical, such as the initial evaluation of suspected acute stroke. In this context, the limited sensitivity observed for small hemorrhagic lesions may represent a relevant limitation, as accurate exclusion of hemorrhage is essential for treatment decisions. Further studies are required to determine whether ULF-pMRI can reliably support clinical decision-making in these settings.

Although ULF-pMRI offers important practical advantages such as bedside availability, its current diagnostic performance is insufficient for the reliable detection of petechial hemorrhagic transformation in acute ischemic stroke, limiting its utility for therapy-relevant clinical decisions, particularly regarding the timing of initiation of antithrombotic or anticoagulant therapy, which depends on the presence and extent of hemorrhagic transformation.

## Limitations

This study has several limitations that should be considered when interpreting the findings. First, the retrospective design may introduce selection bias and limit control over imaging timing and clinical decision pathways. Second, the sample size was relatively small, reducing statistical power, particularly for subgroup analyses and rare hemorrhage patterns. Third, the cohort included a limited case number with space-occupying or clinically significant hemorrhages, which restricts conclusions about the ability of ULF-pMRI to detect larger or more complex bleeding types. Furthermore, the study was conducted at a single center, which may limit generalizability to other institutions with different workflows or patient populations.

## Conclusion

Our results suggest that while ULF-pMRI offers practical advantages such as bedside availability, its current capability in detecting hemorrhagic infarct transformation in acute ischemic strokes is limited. Reliable image-based clinical decisions such as initiation of anticoagulants or antithrombotics in acute ischemic stroke patients should consider standard MRI or conventional CT imaging as preferable imaging modality.

## Data Availability

No datasets were generated or analysed during the current study.
